# Patient-Reported Treatment Experience with Oral Rivaroxaban: Results from the Noninterventional XALIA Study of Deep-Vein Thrombosis

**DOI:** 10.1055/s-0038-1641679

**Published:** 2018-04-11

**Authors:** Stefan Cano, Lorenzo Mantovani, Kerstin Folkerts, Martin Gebel, Kurtulus Sahin, Elizabeth Zell, Danja Monje, Jonas Schneider, Martin van Eickels, Sylvia Haas, Reinhold Kreutz, Walter Ageno, Alexander G. G. Turpie

**Affiliations:** 1 Modus Outcomes, Letchworth Garden City, United Kingdom; 2 CESP-Center for Public Health Research, University of Milan Bicocca, Monza, Italy; 3 Bayer AG, Wuppertal, Germany; 4 ClinStat GmbH, Cologne, Germany; 5 Stat-Epi Associates, Inc., Ponte Vedra Beach, Florida, United States; 6 Bayer AG, Berlin, Germany; 7 Vascular Centre, Munich, Germany; 8 Institute of Clinical Pharmacology and Toxicology, Charité - Universitätsmedizin Berlin, Freie Universität Berlin, Humboldt-Universität zu Berlin, and Berlin Institute of Health, Berlin, Germany; 9 Department of Clinical and Experimental Medicine, University of Insubria, Varese, Italy; 10 Department of Medicine, Hamilton Health Sciences, Hamilton, Ontario, Canada

**Keywords:** ACTS, anticoagulation, patient experience, rivaroxaban, venous thromboembolism

## Abstract

For venous thromboembolism (VTE) treatment, patient satisfaction was shown to improve with rivaroxaban versus standard anticoagulation in the phase III EINSTEIN DVT and EINSTEIN PE trials. This substudy of the prospective, noninterventional XALIA study of rivaroxaban for deep-vein thrombosis treatment assessed if this was also observed in routine clinical practice. Patients enrolled in XALIA who received rivaroxaban or standard anticoagulation treatment were eligible for inclusion in this substudy. Treatment decisions were at the physician's discretion. Patients completed the 17-item Anti-Clot Treatment Scale (ACTS, comprising a 12-item Burdens subscale, a 3-item Benefits subscale and one global item per subscale) during follow-up. The propensity score-matched set (PMS) was used for the main analysis; the adjusted safety analysis (ASAF) set was used for confirmatory purposes. Analyses by follow-up visit and subgroup, including age, sex, and previous VTE, were also conducted. The PMS-ACTS analysis included 458 rivaroxaban-treated and 434 standard anticoagulation-treated patients. Baseline demographic and clinical characteristics were generally similar across treatment arms. ACTS Burdens scores significantly improved with rivaroxaban versus standard anticoagulation (least-squares mean difference of 2.4 ± 0.4 points; *p* < 0.0001); ACTS Benefits scores were numerically higher with rivaroxaban (least-squares mean difference of 0.2 ± 0.1 points; *p* = 0.2). Similar findings occurred across follow-up visits and subgroups. Results were confirmed in the ASAF-ACTS analysis. Consistent with phase III analyses, rivaroxaban was associated with improved ACTS Burdens scores; ACTS Benefits scores numerically favored rivaroxaban, although without reaching statistical significance.

## Introduction

Anticoagulant treatment for venous thromboembolism (VTE), comprising deep-vein thrombosis (DVT) and pulmonary embolism (PE), is associated with various benefits and burdens, some of which are therapy specific. Traditional therapies have several well-known limitations; agents such as low-molecular-weight heparin (LMWH) are administered via injection, which makes self-administration problematic for some patients. 1 Many patients also have a dislike or fear of needles, 2 meaning that repeated injections may not be suitable for them. Vitamin K antagonists (VKAs) require regular anticoagulation monitoring, which contributes to making their use in the outpatient setting suboptimal. A meta-analysis of randomized trials and cohort studies showed that in the first month of treatment, patients were within the target international normalized ratio (INR) range of 2.0 to 3.0 only 56% of the time. 3 Even when the first 3 months of treatment were excluded from the meta-analysis, the time in therapeutic range was only around 75%. The interaction of VKAs with foods containing high levels of vitamin K, other medications, and alcohol contributes to the need for monitoring and management of adverse effects, making their use more burdensome. 4 VKAs have slow and variable onsets of action, and there is considerable variation between individuals in terms of their response to therapy. Other concerns associated with anticoagulation treatment may adversely affect patients' attitudes toward therapy, for example, the perceived risk of bleeding events.

In addition to treatment efficacy, the issues described earlier can impact the patient's subjective impression of the treatment process. Employing measures to assess and quantify parameters such as treatment satisfaction or enhancement of the patient experience is becoming common practice as part of clinical studies, especially as treatment satisfaction has a positive association with adherence and persistence. 5 6 The non-VKA oral anticoagulants (apixaban, dabigatran, edoxaban, and rivaroxaban) can potentially reduce many of the burdens associated with standard therapies. For example, their oral route of administration, combined with a lack of requirement for routine anticoagulation monitoring, would be expected to reduce the burdens associated with repeated injections and INR testing.

The phase III EINSTEIN DVT and EINSTEIN PE studies demonstrated a higher degree of patient treatment satisfaction with rivaroxaban versus standard anticoagulation. 7 8 9 10 The noninterventional XALIA study of rivaroxaban versus standard anticoagulation for the treatment of DVT in routine clinical practice was conducted subsequently. One of the aims of XALIA was to determine whether improved patient experience with rivaroxaban was replicated outside the clinical trial setting; 11 this analysis presents the results of these assessments.

## Materials and Methods

The XALIA study methods are described in the XALIA primary article. 11 The methodology for the Anti-Clot Treatment Scale (ACTS) substudy is presented below.

### Patients

All patients enrolled in XALIA who received rivaroxaban or standard anticoagulation treatment were eligible for inclusion in this substudy. Patients were aged ≥18 years, with objectively confirmed DVT (after approval of rivaroxaban in the PE indication, patients with DVT and concomitant PE were also eligible) and an indication for at least 3 months of anticoagulation treatment. The type, dose, and duration of anticoagulant drug therapy administered to each patient were at the discretion of the attending physician. Patients who received rivaroxaban alone, or who initially received heparin/fondaparinux for a maximum of 48 hours before enrolment, were included in the rivaroxaban cohort, consistent with the approach in the EINSTEIN DVT phase III trial. 7 Patients who received initial heparin or fondaparinux for >2 to 14 days with or without a VKA for 1 to 14 days before switching to rivaroxaban were designated as “early switchers” and were not included in the analysis presented here.

### Anti-Clot Treatment Scale

Patients completed the anticoagulation-specific ACTS during follow-up visits; country-specific and linguistically validated versions of the questionnaire were used. The ACTS comprises 17 items representing negative and positive aspects of anticoagulation treatment: ACTS Burdens (12 scale items plus 1 global item regarding burdens); ACTS Benefits (3 scale items plus 1 global item regarding benefits). Item scores were summed across domains to give an ACTS Burdens score ranging from 12 to 60 and an ACTS Benefits score ranging from 3 to 15, with higher scores in both indicating a more positive patient experience with anticoagulation treatment; scores for global items were not included in the calculations. The ACTS was determined at days 1 to 44 (Visit 1), days 45 to 134 (Visit 2), days 135 to 224 (Visit 3), days 225 to 314 (Visit 4), and any time from day 315 onward (Visit 5). The questions comprising the ACTS questionnaire are shown in the Supplementary Material ( Table S1 ).

### Propensity Score-Adjusted Analysis

Propensity score adjustment was used to address the imbalances in baseline characteristics of the two treatment groups in the safety analysis set. Briefly, analyses were conducted in two ways, in a set of matched pairs as well as stratified by eight homogeneous subclasses, both sets derived with propensity scores. Propensity scores, matched pairs, and subclasses were developed by an independent statistician without knowledge of outcome events and addressed allocation bias via adjustment of the potential effect of unbalanced covariates. 12 13 Matched pairs based on the propensity scores were created using the greedy algorithm. 14 The propensity score derivation and stratified analysis methodologies are included in the Supplementary Material of the XALIA primary article. 11 The main analysis of the ACTS results in this study was conducted on the set of patients with matched propensity scores (the propensity score-matched set [PMS]) who had at least one nonmissing ACTS assessment after implementation of the imputation method (termed the PMS-ACTS set). As a consequence, only a subset of patients consisted of matched pairs. Alternatively, patients with propensity scores that were too high/low were removed from the safety set and eight homogenous subclasses were created for the adjusted safety analysis (ASAF) set. A sensitivity analysis was conducted in the adjusted safety set of patients who had at least one nonmissing ACTS assessment after implementation of the imputation method (ASAF-ACTS).

### Subgroups

Several subgroups were also assessed for differences in ACTS Burdens and Benefits scores between treatments. These subgroups were as follows: age (<60, ≥60 years); body mass index (≤25, >25 to ≤35, >35 kg/m ^2^ ); sex (male, female); hospitalization for index events (yes, no); immobilization (yes, no); language (Dutch, German, French, English, Italian, Swedish, Spanish, Canadian English); provoked VTE (yes, no); reason for treatment (patient's age, patient's living conditions, comorbidities, distance to treating physician, medical or hospital guidelines, availability of drug, price of drug, type of health insurance, other); renal disease (yes, no); active cancer at baseline (yes, no); active cancer at baseline (excluding patients on VKA; yes, no); race (white, black, Asian, not reported); region (western Europe, Canada and Israel, eastern Europe); first available weight (≤70, >70 to <90, ≥90 kg); country (France, Germany, Spain, rest); chronic heart failure (yes, no); diabetes (yes, no); first available creatinine clearance (<30, ≥30 to <50, ≥50 to <80, ≥80 mL/min); patient health insurance (public, fully private, partially private, other [including missing]); cardiovascular disease (yes, no); stroke (yes, no); previous major bleeding event (yes, no); previous VTE (yes, no); thrombophilia (yes, no); venous insufficiency (yes, no); history of hypertension (yes, no); and chronic obstructive pulmonary disease (yes, no).

### Statistical Analysis

The ACTS was completed in accordance with the developer's guidelines. 15 In instances for which more than 50% of questions were missing responses, the scale was considered to be incomplete; partial completion where missing responses were below 50% was addressed by scale-specific mean imputation.

A mixed-model repeated-measures analysis using an unstructured covariance matrix was used to analyze the questionnaire data because the questionnaire responses were multiple measurements on patient experience with treatment over a period of time. The mixed model included ACTS as outcome, and treatment, cancer at baseline, and treatment by visit interaction as covariates. From this model, least square (LS) means for each treatment group and treatment differences and corresponding standard errors (SEs) were presented per visit. The same analysis without treatment by visit interaction for the overall effect with LS means and SEs is shown. For ASAF-ACTS, the LS means were calculated individually per strata and treatment and then combined with a stratified-based combining rule. All statistical significance testing was performed at a two-sided 0.05α level, and all data analysis was done using SAS statistical software package version 9.4 (SAS Institute, Cary, North Carolina, United States).

## Results

### Patients and ACTS Completion Rates

Of the 5,136 patients who received study medication in XALIA, 4,768 (92.8%) were included in the safety analysis set (368 [7.2%] early switchers were excluded from the primary analysis). Subsequently, 253 patients were excluded from the ASAF because they had propensity scores that were either too high or too low. This left an ASAF set of 4,515 patients, 2,505 (55.5%) of who received rivaroxaban and 2,010 (44.5%) who received standard anticoagulation. A total of 1,124 matched pairs (2,248 patients) were included in the PMS set.

Because several patients in XALIA had missing ACTS data, the PMS-ACTS set comprised 892 patients in total, of which 458 (51.3%) received rivaroxaban and 434 (48.7%) received standard anticoagulation. The ASAF-ACTS set included 1,726 patients (1,007 [58.3%] for rivaroxaban, 719 [41.7%] for standard anticoagulation).

### Baseline Demographics and Clinical Characteristics

The baseline demographics and clinical characteristics of the PMS-ACTS set are shown in Table 1 . Patients in the rivaroxaban cohort were generally younger, had higher rates of unprovoked VTE, and had lower rates of renal impairment, concomitant PE, and active cancer than those in the standard anticoagulation group. The baseline characteristics of both analysis sets were similar to those of the full safety analysis set. 11

**Table 1:** Baseline demographics and clinical characteristics of patients in the XALIA treatment satisfaction substudy (PMS-ACTS set)

Characteristic a	Rivaroxaban ( *N* = 458)	Standard anticoagulation b ( *N* = 434)
Mean age, y (SD)	60.1 (15.7)	61.7 (16.6)
Age category		
< 60 y	198 (43.2)	191 (44.0)
≥ 60 y	260 (56.8)	243 (56.0)
Male sex	196 (42.8)	206 (47.5)
Weight		
< 50 kg	4 (0.9)	6 (1.4)
≥ 50 to 70 kg	97 (21.2)	95 (21.9)
> 70 to <90 kg	157 (34.3)	150 (34.6)
≥ 90 kg	119 (26.0)	111 (25.6)
Missing	81 (17.7)	72 (16.6)
First available creatinine clearance		
< 30 mL/min	1 (0.2)	8 (1.8)
≥ 30 to <50 mL/min	20 (4.4)	21 (4.8)
≥ 50 to <80 mL/min	95 (20.7)	77 (17.7)
≥ 80 mL/min	201 (43.9)	160 (36.9)
Missing	141 (30.8)	168 (38.7)
Index diagnosis		
DVT only	409 (89.3)	382 (88.0)
DVT with PE	49 (10.7)	52 (12.0)
Type of VTE c		
Provoked	143 (31.2)	155 (35.7)
Unprovoked	315 (68.8)	279 (64.3)
Previous VTE	124 (27.1)	109 (25.1)
Active cancer at baseline	40 (8.7)	46 (10.6)
Known thrombophilic condition	35 (7.6)	35 (8.1)
Previous major bleeding episode	11 (2.4)	8 (1.8)

Abbreviations: ACTS, Anti-Clot Treatment Scale; DVT, deep-vein thrombosis; PE, pulmonary embolism; PMS, propensity score matched set; SD, standard deviation; VTE, venous thromboembolism.

a *n* (%) unless otherwise stated.

b Standard anticoagulation consisted of initial treatment with unfractionated heparin, low-molecular-weight heparin, or fondaparinux, which could overlap with and be followed by an oral vitamin K antagonist.

c Cancer was not considered when defining DVT as provoked or unprovoked.

For the PMS-ACTS set, the countries with the highest rates of representation were France (30.6%) and Germany (27.2%). A similar pattern was seen with the ASAF-ACTS set, with France and Germany again forming the largest geographical cohorts (30.9 and 29.5%, respectively).

### ACTS Burdens

#### PMS-ACTS Analysis Set

The overall ACTS Burdens score (LS mean difference) was 2.4 ± 0.4 points higher (higher scores indicating a reduced burden) in the PMS-ACTS set in the rivaroxaban group versus the standard anticoagulation group (LS mean scores of 56.1 and 53.7, respectively; *p* < 0.0001), indicative of a significantly reduced burden with rivaroxaban. The higher scores with rivaroxaban were consistent over time, with the LS mean difference in scores ranging from 2.2 (Visits 2 and 4) to 2.9 (Visit 3); the differences were significant at each visit ( Table 2 ).

**Table 2:** ACTS Burdens scores by visit (PMS-ACTS set)

Visit	Rivaroxaban/standard anticoagulation patients ( *n* / *n* ) a	Rivaroxaban/standard anticoagulation scores (LS mean/LS mean)	LS mean difference (SE)	*p* -Value
Overall	458/434	56.1/53.7	2.4 (0.4)	< 0.0001
1	333/295	55.2/52.5	2.7 (0.5)	< 0.0001
2	307/279	56.0/53.8	2.2 (0.5)	< 0.0001
3	179/161	56.1/53.2	2.9 (0.6)	< 0.0001
4	68/75	56.7/54.5	2.2 (0.7)	0.002
5	69/77	56.5/54.1	2.4 (0.6)	0.0002

Abbreviations: ACTS, Anti-Clot Treatment Scale; LS, least squares; PMS, propensity score matched set; SE, standard error.

a ACTS Burdens scores had no effect on treatment discontinuation in the PMS-ACTS analysis set. Patients treated with standard anticoagulation treatment discontinued 1.6 times more than patients treated with rivaroxaban.

#### ASAF-ACTS Analysis Set

The overall ACTS Burdens score (LS mean difference) was 2.4 ± 0.4 points higher in the ASAF-ACTS set in the rivaroxaban group versus the standard anticoagulation group (LS mean scores of 55.2 and 52.9, respectively; *p* = 0.0001), indicative of a significantly reduced burden with rivaroxaban. The higher scores with rivaroxaban were consistent over time, with LS mean difference in scores ranging from 2.0 (Visit 2) to 2.8 (Visit 3); the differences were significantly different at each visit ( Table 3 ).

**Table 3:** ACTS Burdens scores by visit (ASAF-ACTS set)

Visit	Rivaroxaban/standard anticoagulation patients ( *n* / *n* )	Rivaroxaban/standard anticoagulation scores (LS mean/LS mean)	LS mean difference (SE)	*p* -Value
Overall	1,007/718	55.2/52.9	2.4 (0.4)	0.0001
1	743/475	54.6/52.3	2.5 (0.5)	0.0003
2	656/450	55.1/53.2	2.0 (0.5)	0.001
3	371/246	55.2/52.9	2.8 (0.5)	0.0004
4	166/120	55.6/53.5	2.7 (0.8)	0.009
5	155/115	55.4/52.8	2.7 (0.8)	0.002

Abbreviations: ACTS, Anti-Clot Treatment Scale; ASAF, adjusted safety analysis; LS, least squares; SE, standard error.

#### Subgroups

**Fig. 1:**
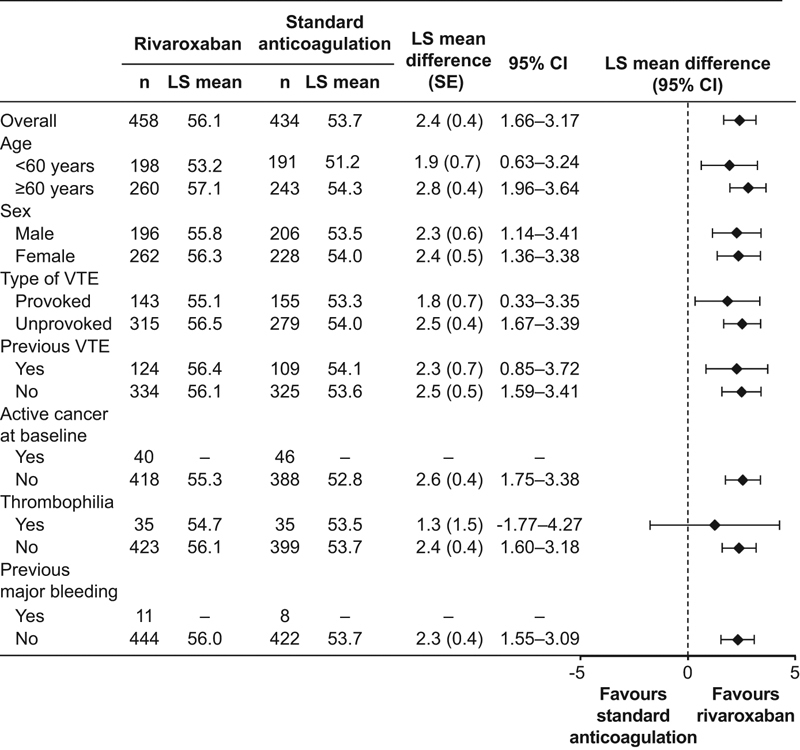
ACTS Burdens score least squares mean differences by subgroup (PMS-ACTS analysis set). Subgroups with missing values had too few patients to enable LS mean difference to be calculated. ACTS, Anti-Clot Treatment Scale; CI, confidence interval; LS, least squares; PMS, propensity score-matched set; SE, standard error; VTE, venous thromboembolism.

Almost all patient subgroups in the PMS-ACTS and ASAF-ACTS sets showed statistically significant differences in ACTS Burdens scores between treatment groups in favor of rivaroxaban, both for overall scores and by treatment visit. Treatment comparisons for selected subgroups are shown in Fig. 1 .

### ACTS Benefits

#### PMS-ACTS Analysis Set

The overall ACTS Benefits score (LS mean difference) was 0.2 ± 0.2 points higher in the PMS-ACTS set in the rivaroxaban group versus the standard anticoagulation group, although this difference was not significant (LS mean scores of 12.1 and 11.9, respectively; *p* = 0.2). The similar ACTS Benefits scores between the two treatment groups were consistent over time, with the LS mean difference in scores ranging from –0.1 (Visit 1) to 0.5 (Visit 2; Table 4 ).

**Table 4:** ACTS Benefits scores by visit (PMS-ACTS set)

Visit	Rivaroxaban/standard anticoagulation patients ( *n* / *n* ) a	Rivaroxaban/standard anticoagulation scores (LS mean/LS mean)	LS mean difference (SE)	*p* -Value
Overall	450/430	12.1/11.9	0.2 (0.2)	0.2
1	326/286	11.6/11.6	–0.1 (0.2)	0.8
2	298/277	12.0/11.4	0.5 (0.2)	0.01
3	177/159	12.1/11.9	0.2 (0.3)	0.4
4	68/75	12.2/12.1	0.1 (0.4)	0.8
5	67/77	12.3/12.2	0.1 (0.3)	0.8

Abbreviations: ACTS, Anti-Clot Treatment Scale; LS, least squares; PMS, propensity score matched set; SE, standard error.

a ACTS Benefits scores showed an odds ratio of 0.91 (95% CI: 0.84–0.98) for treatment discontinuation in the PMS-ACTS analysis set; therefore, the lower the ACTS Benefits score was, the more patients tended to discontinue from the study.

#### ASAF-ACTS Analysis Set

The overall ACTS Benefits score (LS mean difference) was 0.2 ± 0.1 points higher in the ASAF-ACTS set in the rivaroxaban group versus the standard anticoagulation group, although this difference was not statistically significant (LS mean scores of 11.9 and 11.8, respectively; *p* = 0.4). The similar ACTS Benefits scores between the two treatment groups were consistent over time, with the LS mean difference in scores ranging from <0.1 (Visit 5) to 0.2 (Visits 1, 3, and 4; Table 5 ).

**Table 5:** ACTS Benefits scores by visit (ASAF-ACTS set)

Visit	Rivaroxaban/standard anticoagulation patients ( *n* / *n* )	Rivaroxaban/standard anticoagulation scores (LS mean/LS mean)	LS mean difference (SE)	*p* -Value
Overall	989/712	11.9/11.8	0.2 (0.1)	0.4
1	724/465	11.8/11.6	0.2 (0.2)	0.5
2	639/447	11.8/11.6	0.1 (0.2)	0.3
3	365/244	11.9/11.8	0.2 (0.3)	0.6
4	165/119	12.1/11.9	0.2 (0.4)	0.7
5	152/114	12.2/11.9	<0.1 (0.4)	0.5

Abbreviations: ACTS, Anti-Clot Treatment Scale; ASAF, adjusted safety analysis; LS, least squares; SE, standard error.

#### Subgroups

**Fig. 2:**
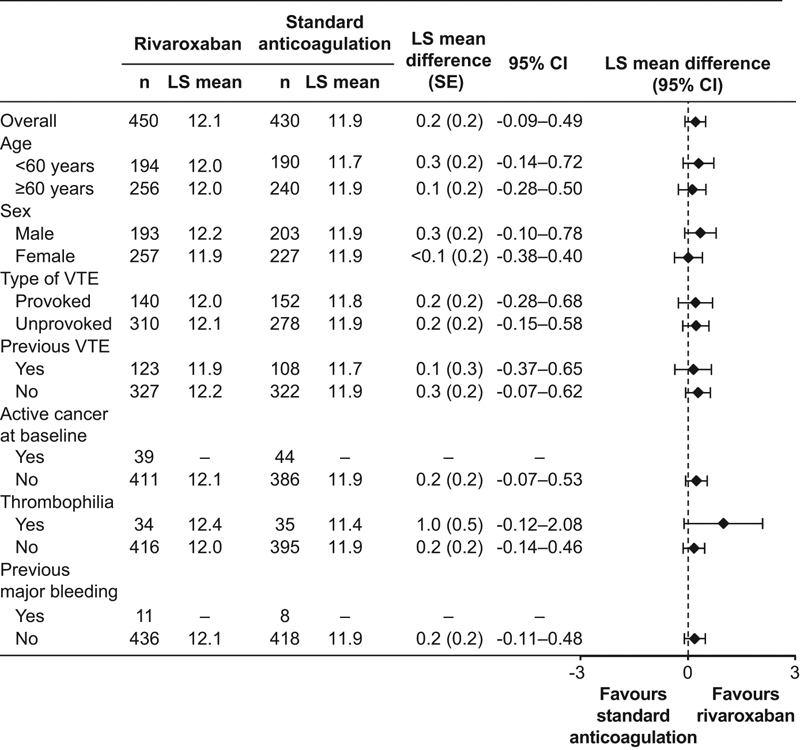
ACTS Benefits score least squares mean differences by subgroup (PMS-ACTS analysis set). Subgroups with missing values had too few patients to enable LS mean difference to be calculated. ACTS, Anti-Clot Treatment Scale; CI, confidence interval; LS, least squares; PMS, propensity score-matched set; SE, standard error; VTE, venous thromboembolism.

As with the total PMS-ACTS and ASAF-ACTS results, the majority of subgroups showed no statistically significant differences between treatment groups for ACTS Benefits scores, either for the overall scores or by treatment visit. For those subgroups that did show differences, almost all were at Visit 2. Treatment comparisons for selected subgroups are shown in Fig. 2 .

## Discussion

The XALIA ACTS substudy compared treatment experience with DVT (also DVT plus concomitant PE after the approval of rivaroxaban in the PE indication) in patients who were treated with rivaroxaban or standard anticoagulation. The results demonstrated that patients given rivaroxaban had an improved treatment experience, both overall and across study time points, in terms of finding the treatment less burdensome than standard anticoagulation. Similarly, the ACTS Benefits scores showed a numerical trend in favor of rivaroxaban. These findings for the ACTS Burdens scores are consistent with results from the ACTS substudy of the EINSTEIN DVT trial. 9 The PMS-ACTS and ASAF-ACTS analysis sets were similar to the overall XALIA safety analysis set in respect of baseline demographic and clinical characteristics. The exceptions to this were that patients in the PMS-ACTS set were on average ∼1 year older (60.9 vs. 59.8 years) and had higher rates of previous VTE (26.1 vs. 23.3%) than patients in the safety analysis set.

The completion rates of the ACTS for the rivaroxaban and standard anticoagulation groups in the ACTS substudy in XALIA were consistent with that observed for EINSTEIN DVT, where around 40% of all patients were analyzed in the treatment satisfaction substudy. Similar to EINSTEIN DVT, the open-label nature of treatment administration in XALIA meant that patients were able to evaluate the impact of the therapy directly because they were not given dummy controls or subject to sham INR monitoring. In addition, the real-world nature of the XALIA study also means that the findings are reflective of actual clinical practice. Subgroup analysis by baseline patient demographics and clinical characteristics revealed a similar pattern to the main analyses for ACTS Burdens and Benefits scores. For the ACTS Burdens scores, most subgroups showed significant between-treatment differences favoring rivaroxaban for overall scores and scores by visit. Of note, the effect size was smaller or showed a marginal trend toward standard anticoagulation in patients with provoked VTE; a more robust follow-up and health service contact may explain the almost equivalent response to rivaroxaban. In addition, the ACTS Burdens scores did not show a statistically significant between-treatment difference in patients with thrombophilia; again, a more intensive follow-up may explain this finding. For ACTS Benefits scores, there was a numerical trend favoring rivaroxaban across visits and subgroups, although only a limited number of subgroups showed statistically significant differences, and these were almost all at Visit 2.

A limitation of this study is that it assessed only patient experience over a relatively short period of time (the median treatment duration in XALIA was 181 days with rivaroxaban and 190 days with standard anticoagulation); therefore, it would also be of interest to determine whether the patient experience findings observed in XALIA persist in the longer term. It is important to note that the ACTS questionnaire is a specific measure that focuses on all medical aspects of the treatment experience that are important to patients and gathers information on all aspects of anticoagulation therapy, both positive and negative. 9 Therefore, a widely used, generic measure (e.g., the Treatment Satisfaction Questionnaire for Medication version II [TSQM II]) focusing on the medication per se could complement the ACTS results. 9 16 It is often highlighted that the use of two measures offers several advantages: although generic measures allow comparisons of outcomes across different study populations, thus enhancing the generalizability of findings, specific measures may be more sensitive for the detection and quantification of small changes that may be important to physicians or patients. 17

Another limitation of the study was that the patient population was constrained by the availability of ACTS values; the ASAF-ACTS contained only 38% of the ASAF patients resulting in 1,726 patients from eight countries (Canada, Denmark, England, France, Germany, Italy, Spain, and Sweden); therefore, caution is advised when extrapolating these results to the wider patient population.

In conclusion, despite the limitations described earlier, this substudy confirms the findings from the EINSTEIN phase III studies that the patient experience is less burdensome with rivaroxaban treatment than with standard anticoagulation. This finding was expected based on the characteristics of the two therapies. The differences in ACTS Benefits scores were numerically in favor of rivaroxaban versus standard anticoagulation treatment in XALIA, although this did not reach statistical significance. These findings may potentially impact on factors such as treatment adherence and persistence and could, therefore, positively impact patients who have a long-term requirement for anticoagulation treatment.
