# Supplementary material for: Patient-Reported Treatment Experience with Oral Rivaroxaban: Results from the Noninterventional XALIA Study of Deep-Vein Thrombosis
Source: TH Open. 2018 Apr 11;2(2):e139–46. doi: 10.1055/s-0038-1641679 (PMC6524861; doi:10.1055/s-0038-1641679)
Supplement: Supplementary file 1 — Supplementary Material [file 10-1055-s-0038-1641679-s180011.pdf]

Supplementary Material

In the ACTS questionnaire (►**Table S1**), patient experience with anticoagulation treatment is ranked on a 5-point Likert scale from “Not at all” to “Extremely.” The first 12 items of ACTS (Burdens) use a reverse scoring system from 5 (not at all) to 1 (extremely), whereas the last three items of ACTS (Benefits) are scored from 1 (not at all) to 5 (extremely).

Table S1 The ACTS questionnaire[15]

Item	Not at all	A little	Moderately	Quite a bit	Extremely
Bleeding/vigorous activities					
Bleeding/usual activities					
Bruising					
Avoid other medicines					
Limit eat/drink					
Hassle/daily					
Hassle/occasional					
Difficult to follow your anticoagulation treatment					
Time-consuming anticoagulation treatment					
Worry about anticoagulation treatment					
Frustrating anticoagulation treatment					
Burden of anticoagulation treatment					
Confident in anticoagulation treatment					
Reassured by anticoagulation treatment					
Satisfied with anticoagulation treatment					
